# Neurotoxicity of Engineered Nanomaterials: Testing Considerations

**DOI:** 10.3389/fpubh.2022.904544

**Published:** 2022-07-13

**Authors:** Eleonora Scarcello, Adriana Sofranko, Tina Wahle, Roel P. F. Schins

**Affiliations:** IUF - Leibniz Research Institute for Environmental Medicine, Düsseldorf, Germany

**Keywords:** neurotoxicity, nanomaterials, *in vitro*, toxicokinetic, engineered nanomaterials

## Abstract

As with toxicology in general, major challenges have emerged in its subfield neurotoxicology regarding the testing of engineered nanomaterials (ENM). This is on the one hand due to their complex physicochemical properties, like size, specific surface area, chemical composition as well as agglomeration and dissolution behavior in biological environments. On the other hand, toxicological risk assessment has faced an increasing demand for the development and implementation of non-animal alternative approaches. Regarding the investigation and interpretation of the potential adverse effects of ENM on the brain, toxicokinetic data are relatively scarce and thus hampers dose selection for *in vitro* neurotoxicity testing. Moreover, recent *in vivo* studies indicate that ENM can induce neurotoxic and behavioral effects in an indirect manner, depending on their physicochemical properties and route of exposure. Such indirect effects on the brain may proceed through the activation and spill-over of inflammatory mediators by ENM in the respiratory tract and other peripheral organs as well *via* ENM induced disturbance of the gut microbiome and intestinal mucus barrier. These ENM specific aspects should be incorporated into the ongoing developments of advanced *in vitro* neurotoxicity testing methods and strategies.

## Introduction

The steady development and production of engineered nanomaterials (ENM) and their expanding range of uses in various fields requires an appropriate assessment of their potential health effects in humans. Initial concern about the harmful effects of ENM emerged from: (i) early inhalation toxicology studies with specific types of ultrafine particles, showing increased local pulmonary toxicity compared to larger particles of the same chemical composition, and (ii) studies that revealed evidence of increased uptake and translocation of nanoscale particles to tissues and organs beyond the initial deposition site in the respiratory tract (1, 2). The importance of toxicology research on ENM has been substantiated by studies that support a role of ambient ultrafine particles (UFP) in air pollution-associated diseases (3). Similarly, increased attention has been given to the potential neurotoxicity of ENM (4–6). A pioneering inhalation study by Oberdoerster and co-workers (7), demonstrated that insoluble carbon particles in the nano size range can rapidly translocate to the brain upon deposition in the nasal mucosa in rats. This observation formed a major trigger for further research to determine potential adverse health effects of inhaled UFP and ENM on the brain.

Adverse effects on the brain have been shown nowadays in various studies. Investigations with diesel engine exhaust (DEE), collected diesel exhaust particles (DEP) or ambient particulate matter (PM) (6, 8) as well as on-site-exposure studies with concentrated ambient PM (CAPs) have provided important support for the growing number of epidemiological studies that link air pollution to neurological diseases including dementia (9, 10). Neurotoxicological studies with ENM in rodents have been performed with the most widely produced and used materials like Ag, SiO_2_, TiO_2_, CeO_2_, ZnO and carbon black. However, also less common materials have been studied like gold, quantum dots or iridium nanoparticles (11–13).

In the context of regulatory testing, neurotoxicity of ENM can be defined as a direct or indirect adverse effect caused by such particulate materials on the structure or functioning of the nervous system (14, 15). The scope of this mini-review is not to provide an in-depth overview of all studies that have been performed with various types of ENM and to provide a state of the art on their identified underlying mechanisms of neurotoxicity. For this we refer to various reviews by others [e.g. (5, 16, 17)]. In this paper, we highlight some of the unique physicochemical properties and associated effects of ENM that should be considered during testing and interpretation of their potential neurotoxicity.

In general, as with other effects and associated disease outcomes (e.g., in the respiratory or cardiovascular system), oxidative stress and inflammation are also considered key processes of potential neurotoxic and neurological consequences following ENM exposure (5, 6). Oxidative stress in ENM exposed cells, can cause activation of redox-sensitive signaling cascades involved in activation of pro-inflammatory cytokines and chemokines, proliferation, apoptosis and DNA damage induction (18), representing mediators or processes that all have been implicated in neurological and neurodegenerative diseases (5, 19). When taken together, current available neurotoxicological studies with ENM suggest substantial differences in hazards, strongly depending on their physicochemical properties, including the chemical composition. However, as we will discuss later, such an interpretation must take into account specific differences in dosimetry, (i.e., dose and route of administration) as well as the species selected for investigation (e.g., rat vs. mouse) used in the various studies, in perspective to realistic human exposures.

## Neurotoxicity-Relevant Exposure Routes of Enm

Originally, nanotoxicology research has strongly focused on the evaluation of the potential adverse health effects that involve the inhalation route of exposure and pulmonary health risks. This obviously relates to the principal relevance of occupational exposures during manufacturing and handling of ENM. The emerging consensus that short- and long-term exposure to ambient PM is a risk factor for cardiovascular disease (20) has been a main drive to initiate studies that explored cardiovascular effects and underlying mechanisms of action for ambient UFP as well as for various types of ENM *via* inhalation exposure (20–22). Regarding this exposure route, for neurological evaluations, the importance of translocation of nasally deposited particles to the brain is now widely recognized. Herein, it is proposed that ENM can reach the olfactory bulbs of the brain following deposition on the nasal olfactory epithelium and subsequent translocation along the olfactory nerve (7). However, various additional routes of translocation into the brain should be acknowledged. In the upper respiratory tract, this may for instance also include translocation *via* the trigeminal nerve, while alveolar deposited ENM may enter the brain upon their translocation into the circulation (6). In contrast to the neuronal routes, this latter pathway also requires a subsequent passage of the blood-brain barrier (BBB).

The relative importance of translocation from the upper *vs*. lower respiratory tract has been elegantly explored in inhalation studies using radiolabelled iridium nanoparticles in rats and revealed the significance for both deposition sites (23). However, it should be emphasized that the significance of these various translocation routes may largely differ between various types of ENM depending on their composition and physicochemical properties and the associated impact of differently absorbed proteins and lipids (corona) on their translocation kinetics (6). Obviously, regarding the deposition and subsequent rate of translocation of nano-sized particles from the respiratory tract system, the distinct anatomical and physiological differences between rodents and humans must be taken into account as well (19).

**Figure 1:**
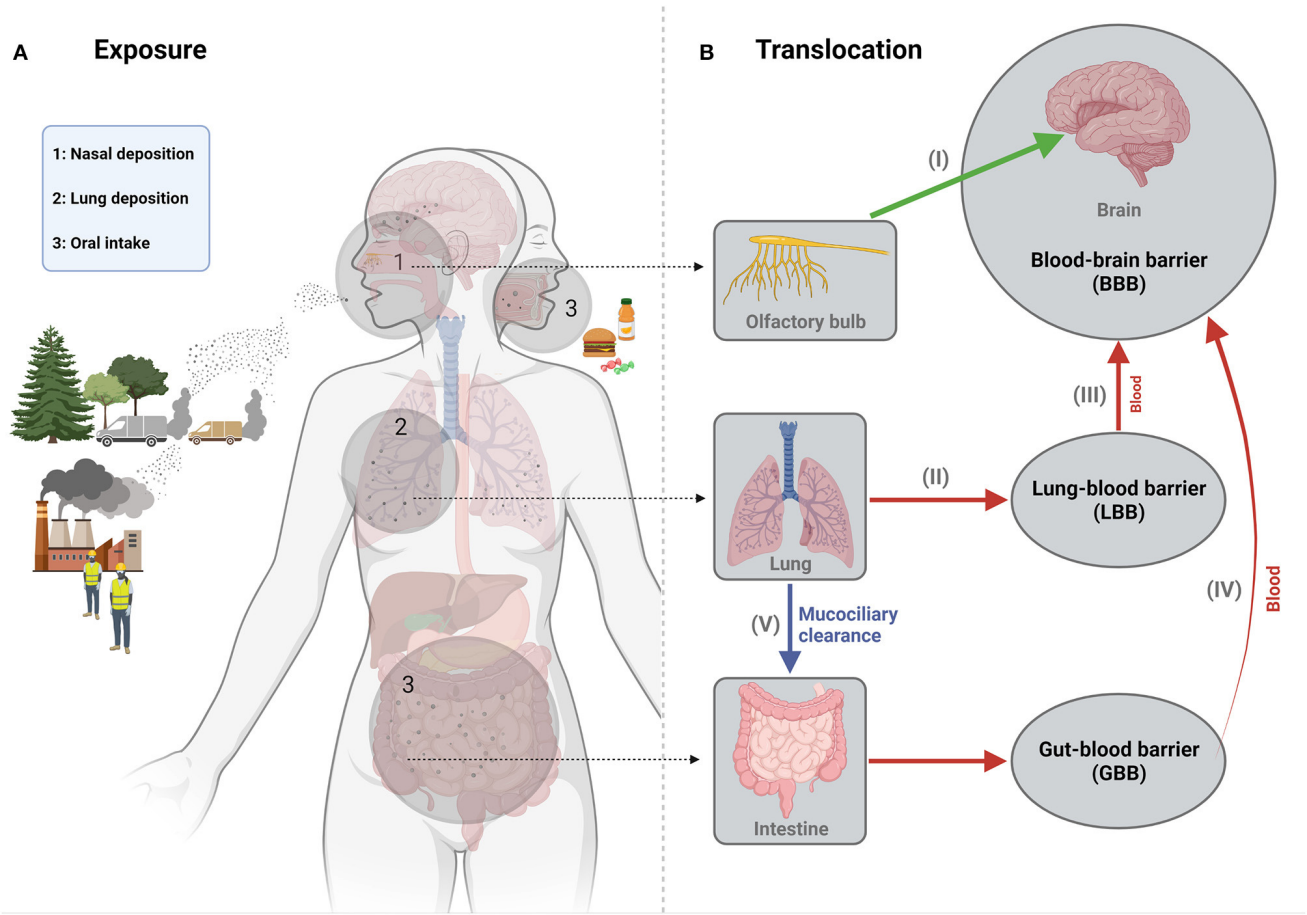
Schematic representation of the main exposure **(A)** and translocation **(B)** routes of engineered nanomaterials in relation to their potential neurotoxicity. Concerns about the neurotoxicity of nanoparticles via inhalation exposure exist in particular for UFP originating from combustion processes at transport-dominated locations. Inhalation represents the most important occupational exposure route for ENM, during their manufacturing or further processing. The most relevant pathway of translocation to the brain for such inhaled particles involves their uptake and retrograde transport along the olfactory nerve upon deposition in the nasal cavity (I). UFP and ENM deposited in the lower respiratory tract can also translocate from this region into the bloodstream upon crossing the “lung-blood barrier (LBB)” (II) and a subset of these particles may thus enter the brain parenchyma across the blood-brain barrier (III). In non-occupational settings, the oral exposure route is particularly relevant for ENM, which may be present in food as additives or contaminants. Uptake across the intestinal mucosal barrier into the bloodstream (IV) of these ENM may thus also result in translocation into the brain. This pathway should also be considered for the fraction of inhaled small particles that is swallowed upon lung mucociliary clearance (V). Depending on exposure levels and their physicochemical properties, accumulation of ENM in the brain may thus directly affect brain structures and cells. However, neurotoxicity and neurological disturbances may also proceed in an indirect manner, for instance, driven by inflammatory effects of inhaled or ingested ENM at the organ of entrance or by ENM induced gut microbiome dyshomeostasis. Figure created with Biorender.com.

While inhalation is the primary exposure route of interest for most bulk-manufactured ENM in occupational settings, other routes should also be considered regarding potential neurotoxicity. Systemic availability *via* dermal exposure, either occupationally or, for example, through its presence in cosmetics, is unlikely to be a major concern for neurological disease risks. In terms of dosimetry and accumulation into the brain, dermal uptake has been generally considered as negligible (2) although this may differ for instance in relation to impaired skin conditions during occupational exposure (24). Exposure to ENM *via* the oral route can be considered much more relevant and has therefore also become a subject of increasing interest in neurotoxicology research. Concerns about the adverse health effects of ingested ENM have increased with their growing number of applications in nanomedicine and particularly in the food sector, where they are used, for example, as food additives or incorporated in food packaging (25, 26). Accumulation of ENM in the brain following oral uptake is envisaged by their successive translocation from the intestine into the systemic circulation and BBB passage. This therefore also represents a potential translocation route for the fractions of inhaled UFP and ENM that are swallowed following mucociliary clearance (1, 27). Translocation from the intestine has indeed been demonstrated for specific ENM of poor solubility in rodents. However, quantitative findings by and large indicate that the amount of accumulation into the brain following oral exposure may be minimal to absent as well [e.g., (28–30)]. A schematic outline of the various exposure and translocation routes that may result in accumulation of UFP and ENM in the central nervous system is shown in Figure 1.

## Neurotoxicity Evaluation of ENM

Neurotoxicity of ENM has been explored in rodent studies as well as in a rapidly growing number of *in vitro* studies [e.g., see reviews (5, 16, 17)]. While neurotoxicology research involves the investigation of effects of toxicants on both the central and peripheral nervous system (14, 15), current ENM research has strongly focused on the brain. Taken together, current available literature indicates that various ENM may have neurotoxic potential. However, studies cannot always be interpreted unambiguously, not least because of the unique physicochemical properties and behavior of the investigated nanomaterial in biological environments. Since the importance of research into the potential toxicity of ENM was recognized, large-scale research initiatives have been launched to dedicate research to support and improve their risk assessment. For instance, the extensive OECD Testing Programme of Manufactured Nanomaterials was launched, among others, to explore to what extent existing strategies and methods for the safety testing of chemicals are also directly applicable to ENM (31). Over the years, a vast array of studies, have been devoted to the investigation of the toxicity of various ENM, including large-scale interdisciplinary research projects. In this context, several assay artifacts could be identified when testing ENM that could lead to false negative or positive data. As a result, specific assay modifications were developed and further testing recommendations were introduced to minimize potential misclassification [e.g., (32)]. It is crucial that such modifications also be deployed in current available *in vitro* neurotoxicity assays as well as in early stage development of novel test strategies in this research field (33, 34).

In Table 1, we have summarized intrinsic characteristics and properties of ENM in biological systems that may affect neurotoxicity assay outcomes as well as approaches that can be used to avoid such assay artifacts and potential misclassification. Above all, the reliability of *in vitro* testing approaches for neurotoxicity relies heavily on the use of appropriate ENM dispersion protocols which have been developed over many years for *in vitro* testing in general [e.g., (35, 36)]. Neurotoxicity testing should also incorporate the necessary control experiments to ensure that assay readouts are not disturbed due to interference of ENM with assay components such as, for instance, by adsorption of dyes, inactivation of reagents or scattering or quenching of (fluorescence) light. Approaches for such control experiments have been proposed and described by various investigators [e.g., (36–38)] and can be used as a basis when developing novel neurotoxicity assays. A particularly great progress in improving *in vitro* assays for ENM has been made following the recognition of the role of the protein/lipid corona in the toxicity of ENMs. In addition to the aforementioned establishment of the importance of the corona in the toxicokinetics of ENMs, its demonstrated influence on cellular uptake and toxicity has been used for further assay adaptations. Specific *in vitro* protocols nowadays incorporate a pre-coating step of the pristine ENM with e.g., (lung) surfactants or serum, or include digestion simulation protocols with gastrointestinal model fluids, to better reflect “bio-nano” interactions that are considered to take place *in vivo* depending on the route of exposure (39, 40).

**Table 1:** ENM neurotoxicity testing considerations.

	**ENM characteristics and properties**	**Considerations and recommendations**	**References**
***In vitro assays***	Effects of agglomeration status and dissolution rate on ENM toxicity	Use of standardized dispersion protocols; characterization of the physicochemical properties of ENM in test environment (e.g., cell culture medium)	(35, 36)
	Interferences with *in vitro* assays (e.g., adsorption and/or inactivation of assay reagents, disturbance of assay readouts by quenching, auto-fluorescence)	Inclusion of non-particulate assay controls; testing of adsorption quenching using ENM spiking at different concentrations	(36–38)
	Formation and alteration of “corona” upon ENM entrance and distribution in biological systems; associated alterations in toxicokinetic and toxicodynamic properties	Testing of pristine ENM vs. ENM (pre)treated with “corona” mimicking compounds and further ENM-surface modifying environments, e.g., (model) lung surfactant, serum proteins, artificial digestion fluids (stomach, intestine)	(39, 40)
	Dosimetry aspects	Use input from toxicokinetic/PB-PK modeling studies for *in vitro* dosing justification and outcome interpretation	(25, 41)
***In vivo assays***	Effects of exposure route (inhalation, ingestion) and selected application method on physicochemical properties of ENM	Critical evaluation of the limitations and potential flaws by non-physiological (bolus) administration of ENM, i.e.,: -Inhalation vs. intranasal instillation, intratracheal instillation, pharyngeal aspiration -Application in drinking water or food vs. oral gavage	(42, 43)
	Toxic effects of ENM on entrance organs, e.g., induction of lung inflammation, disturbance or gut homeostasis	Evaluation of inflammation, oxidative stress and barrier integrity effects for organ of entrance (respiratory tract, gastrointestinal tract); analyses of ENM effects on microbiome	(5, 6, 27, 44, 45)

Interpretation of neurotoxicity findings with ENM from *in vitro* studies will also benefit from improved dosimetry considerations. The strength of outcomes of *in vitro* studies increases with the selection of relevant test concentration ranges that are guided by outcomes from toxicokinetic investigations in rodents (25, 41), and support human health risks estimations under realistic exposure scenarios. Indeed, concentrations of ENM applied in neurotoxicity tests are often in the same order of magnitude as those used for *in vitro* investigations of effects at entrance organs (i.e., lung, intestine), while available toxicokinetic studies indicate large differences in gradients of locally achieved doses. Unfortunately, in-depth quantitative toxicokinetic studies and physiologically based pharmacokinetic (PBPK) modeling studies with ENM in rodents are currently still scarce [e.g., (30, 46–48)]. Moreover, such investigations are typically limited to one or few specific (model) compounds that may not be representative for other types of ENM, in view of each material's unique physicochemical properties. Common quantitative analytical methods used in toxicokinetic studies include AAS and ICP-MS. However, these methods cannot distinguish particulate from non-particulate (e.g., dissolved) compounds. Approaches that do allow for appropriate morphological and size-resolved analyses like electron microscopy are merely qualitative and thus may have limited relevance in toxicokinetic studies. However, these methods can provide important information to complement *in vitro* data. For instance, if a toxicokinetic investigation with a metal-based ENM would reveal exclusive accumulation of dissolved metal in the brain, *in vitro* neurotoxicity studies with the pristine particulate material would be irrelevant and redundant.

*In vitro* neurotoxicity studies can contribute to hazard identification and are nowadays increasingly used and further developed with high-content and high-throughput adaptations to further reduce animal studies (5, 49). Yet, rodent studies currently still remain a major component of neurotoxicity risk assessment. They can simultaneously tackle multiple aspects of neurotoxicity *via* evaluation of neurophysiological, neurochemical, neuroanatomical and behavioral endpoints (8). *In vivo* neurotoxicity of ENM can, for instance, be investigated along the design recommendations of the OECD Test Guideline TG 424 (“Neurotoxicity Study in Rodents”) or embedded in subacute/subchronic oral toxicity studies (e.g., TG 407, 408) or inhalation toxicity studies (e.g., TG 412, 413) by inclusion of specific biochemical and molecular markers of neurotoxicity or neurobehavior tests. Apart from the selection of dose range and exposure duration, also the method of administration of ENM is important for the interpretation of the *in vivo* neurotoxicity findings. For instance, instead of inhalation exposures, studies have used intranasal administration with ENM at very high doses that do not reflect realistic deposition kinetics during inhalation exposure (6, 23). Such intranasal application in terms of dosimetry can exaggerate the neurological impact of ENM *via* the olfactory route, e.g., as a result of local damage and tissue impairment. Conversely, pharyngeal aspiration and intratracheal instillation studies bypass the nasal compartment. While these respective approaches can be very useful for specific mechanistic evaluations including toxicokinetic studies, they are obviously of lower relevance for quantitative risk assessment compared to inhalation studies.

Along the same lines, oral exposure studies with ENM also can involve various methods of administration that may have a large impact on toxicokinetics and toxicodynamics and thus study outcome interpretation. The most commonly applied method of oral administration is gavage. However, like the methods of intranasal instillation, intratracheal instillation and pharyngeal aspiration for the inhalation route, this represents a bolus dose delivery of ENM. Application of ENM in drinking water or in food thus represent more realistic administration methods. Marked differences in accumulations of (elemental) silver in brain were for instance observed following single oral gavage versus application in food pellets (42). However, also studies that address neurotoxicity upon administration in drinking water or food as “vehicles” require additional interpretation. Application in drinking water may affect dosing as well as the physicochemical properties of ENM as a result of agglomeration, sedimentation or dissolution. Introduction in food unavoidably results in complex ENM food matrix interactions that can strongly impact on various physicochemical properties as well including surface-reactivity. Drinking water vs. food applications will also result in different stomach and intestine passage times as well as digestive processes. Accordingly, the selection of the method and regime of application may have major potential impact on neurotoxic effects in animal studies. Various *in vitro* methods have been developed in recent years to simulate how digestion processes can affect the physicochemical properties of ENM (43).

## Indirect Effects of ENM on Neurotoxicity

A final important aspect in neurotoxicity risk assessment of ENM is the consideration of direct vs. indirect effects. On the one hand, it is recognized that *in vivo* neurotoxicity studies may detect indirect effects that are not neurotoxicity-specific. For instance, adverse behavioral responses in exposed rodents may be the consequence of the animals' responses to impairments in other organs and thus a mere reflection of general sickness (8). Inclusion of neurotoxicity-independent indicators of systemic toxicity in the study design, for instance by histology and clinical biochemistry, thus benefits insight on underlying mechanisms of identified neurobehavioural responses in the rodent models and improve judgement of their relevance for neurotoxicity risk in humans. In this context, it is pointed out to be particularly careful when interpreting single-dose animal studies performed at very high dose levels (8). On the other hand, studies may also identify neurotoxicity effects that, albeit indirect, may have a strong mechanistic basis and relevance for humans. The importance of indirect effects of inhaled particles, including ENM, in mutagenesis in the respiratory tract is nowadays well recognized, and considered to be driven by oxidative and proliferative mediators released from recruited inflammatory phagocytes (50, 51). Sustained pulmonary inflammation by ENM has also been proposed as a mechanism for systemic responses e.g., in the cardiovascular system (52). In turn, it is nowadays also increasingly recognized that systemic peripheral inflammation can contribute to neurotoxicity and neurological disease, e.g., *via* activation of microglia by inflammatory cytokines and other mediators or as a result of infiltration of the brain by peripheral immune cells [reviewed by (44)]. Interestingly, these indirect effects can be amplified in conditions of BBB impairments, and effects on the integrity of this barrier and associated neurotoxic responses have indeed been demonstrated for inhaled ENM (53, 54).

Finally, indirect mechanisms of neurotoxicity should also be recognized for ENM in relation to oral exposure. It has emerged that oral exposure to ENM can lead to changes in the intestinal microbiome (45, 55). Intestinal dyshomeostasis resulting from alterations in microbiota composition and their products (e.g., short-chain fatty acids and associated bidirectional gut-brain-axis signaling process that influence mucosal immune responses and the integrity of the intestinal barrier (56, 57). As such, one can even hypothesize that potential effects of inhaled ENM on the brain could involve microbiome-gut-brain axis effects following their mucociliary clearance (27).

## Discussion

With regard to the potential neurotoxic effects of ENM, inhalation and ingestion represent the two most relevant exposure routes. Depending on the route and exposure levels as well as on their specific physicochemical characteristics, ENM may reach the brain *via* various pathways (Figure 1). While available toxicokinetic/PBPK studies generally indicate that translocation to the brain will be low or even absent, this cannot necessarily be extrapolated to all types of ENM. Further research is needed to guide *in vitro* dosimetry of molecular mechanistic and animal-alternative neurotoxicity testing methods as well as to substantiate assessment of translocation and potential accumulation of ENM for the human brain. In contrast to current *in vitro* methods, *in vivo* studies can also identify neurotoxic effects that results from indirect mechanisms of action of ENM, for instance, mediated by pulmonary and systemic inflammatory mediators or altered gut-brain axis signaling (Figure 1).

To avoid misclassification of ENM regarding their potential neurotoxicity, dosimetric and mechanistic aspects discussed in this paper and summarized on Table 1, should be critically considered in the current and future development of alternative testing methods and strategies. In the regulatory context, the assessment of neurotoxicity has traditionally been strongly focused on the evaluation of *in vivo* data, whereas *in vitro* assays have been seen merely as complementary (8, 49). The respective advantages and disadvantages of *in vivo* and *in vitro* tests have been discussed and weighed over the years with respect to their values in human neurotoxicity risk assessment [see, for instance: (58–60)]. The importance of animal testing reduction and replacement methods in toxicology is widely recognized today, both in basic and applied research. The extent to which currently available and newly developed *in vitro* neurotoxicity tests are reliable for the assessment of ENM increases with the recognition and active investigation of the potential occurrence of assay artifacts with these complex particulate substances. At the same time, there is a need for further research into the role of ENM-induced peripheral inflammation, gut dyshomeostasis and other possible indirect mechanisms in the causation of neurological and neurodegenerative diseases.

## Author Contributions

All authors listed have made a substantial, direct, and intellectual contribution to the work and approved it for publication.

## Funding

This project has received funding from the European Union's Horizon 2020 research and innovation programme under grant agreement no. 814978 (TUBE).

## Conflict of Interest

The authors declare that the research was conducted in the absence of any commercial or financial relationships that could be construed as a potential conflict of interest.

## Publisher's Note

All claims expressed in this article are solely those of the authors and do not necessarily represent those of their affiliated organizations, or those of the publisher, the editors and the reviewers. Any product that may be evaluated in this article, or claim that may be made by its manufacturer, is not guaranteed or endorsed by the publisher.
